# Successful Treatment of Postoperative External Biliary Fistula by Selective Nasobiliary Drainage

**DOI:** 10.1155/1992/58436

**Published:** 1992

**Authors:** C. Vagianos, A. Polydorou, T. Karatzas, C. Vagenas, M. Stavropoulos, J. Androulakis

**Affiliations:** 1 Department of Surgery University of Patras. Patras Medical School Patras 265 00 Greece; 2 Endoscopic Unit Department of Surgery Hippocration Hospital Athens Greece

## Abstract

A 25-year old man presented with a high output external biliary fistula after an operation for a giant hydatid cyst of the liver. Endoscopic sphincterotomy was inadequate to close the fistula. A nasobiliary tube was selectively inserted into the leaking hepatic duct and bile was continuously aspirated. The fistula and the residual cavity healed completely. Details of the patients' management using this alternative technique, are discussed.


*HPB Surgery*, 1992, Vol. 6, pp. 115–124
Reprints available directly from the publisher
Photocopying permitted by license only

© Harwood Academic Publishers GmbH
Printed in the United States of America

### CASE REPORT

### SUCCESSFUL TREATMENT OF POSTOPERATIVE EXTERNAL BILIARY FISTULA BY SELECTIVE NASOBILIARY DRAINAGE

C. VAGIANOS[1], A. POLYDOROU[2], T. KARATZAS[1], C. VAGENAS[1], M. STAVROPOULOS[1] and J. ANDROULAKIS[1]

[1]*Department of Surgery, University of Patras. Patras Medical School, Greece and* [2]*Endoscopic Unit, Department of Surgery, Hippocration Hospital, Athens, Greece*

*(Received 8 April 1992)*

A 25-year old man presented with a high output external biliary fistula after an operation for a giant hydatid cyst of the liver. Endoscopic sphincterotomy was inadequate to close the fistula. A nasobiliary tube was selectively inserted into the leaking hepatic duct and bile was continuously aspirated. The fistula and the residual cavity healed completely. Details of the patients' management using this alternative technique, are discussed.

KEY WORDS: Hydatid cyst, biliary fistula, endoscopic sphincterotomy, nasobiliary drainage

### INTRODUCTION

Surgery of hepatic hydatidosis is often complicated by postoperative external biliary fistulae, especially when the residual cavity communicates with large biliary ducts. Rupture of a hydatid cyst into the biliary tract is a common complication of the disease, with an incidence ranging between 5 and 25% in different series[1]. An external fistula may develop following surgical drainage of such a cyst, as higher pressures in the biliary system make it easier for bile to flow into the residual cavity than to the duodenum[2]. Expectant management is advocated initially, as such fistulae usually close spontaneously. Endoscopic retrograde cholangiography is indicated to identify coexisting peripheral obstruction, and as a rule endoscopic sphincterotomy promotes healing[2,3]. Fistulae not responding to endoscopic sphincterotomy have been successfully treated by placing an endoprosthesis[4], by endoscopic embolization of the peripheral bile duct[5] or by reoperation. We report a case of a persistent, high-volume, postoperative biliary cutaneous fistula that developed after surgical drainage of a giant hepatic hydatid cyst. The fistula was successfully treated by an endoscopically placed nasobiliary catheter. This technique provides a simple and safe alternative to reoperation which may be complex in such cases.

Address correspondence to: Constantine E. Vagianos, MD, Department of Surgery, University of Patras Medical School, Patras 265 00 Greece

### CASE REPORT

A 25-year-old man was operated because of a large, multilocular hydatid cyst of the liver (22 cm in greatest diameter) (Figure 1). At surgery the cyst was evacuated,

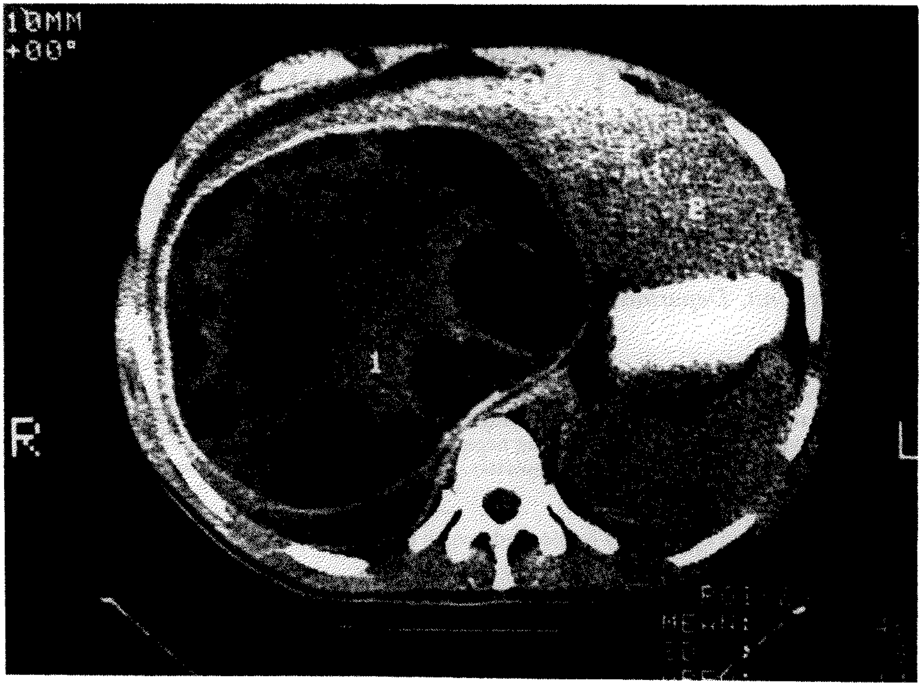

**Figure 1** CT scan of the liver. The right lobe has been largely replaced by the giant hydatid cyst.

sterilized with hypertonic saline and drained with a high vacuum, closed system drain (Redon Ch. 16, Medinorm, Germany). The postoperative course was complicated by a biliary cutaneous fistula, yielding 1500 ml per day through the drain. Conservative management was used first, but as the volume of bile drained did not decrease, endoscopic retrograde cholangiography was performed on the 30th postoperative day. It revealed no peripheral obstruction, but four major leaks (three from the right and one from the left biliary system) and a large intrahepatic assumulation of the contrast material in the residual cavity (Figure 2). An endoscopic sphincterotomy was performed, using a PT-30 (Wilson Cook) sphincterotome. The bile leak decreased to 1000 ml/day and remained constant. A further cholangiogram, forty days later, showed that the leak from the left duct had closed, the cavity had decreased slightly in size and the three leaks from the right duct remained unchanged. A 7 French, 250-cm-long, nasobiliary polyethylene tube (ENBD-7-Liguary, Wilson Cook) was inserted selectively into the right hepatic duct with the tip above the leaks (Figure 3). Bile was aspirated continuously through the tube with a 20 cm $H_2O$ negative pressure, yielding an average of 400 ml bile/day. Biliary drainage from the fistula decreased dramatically, ten days later the

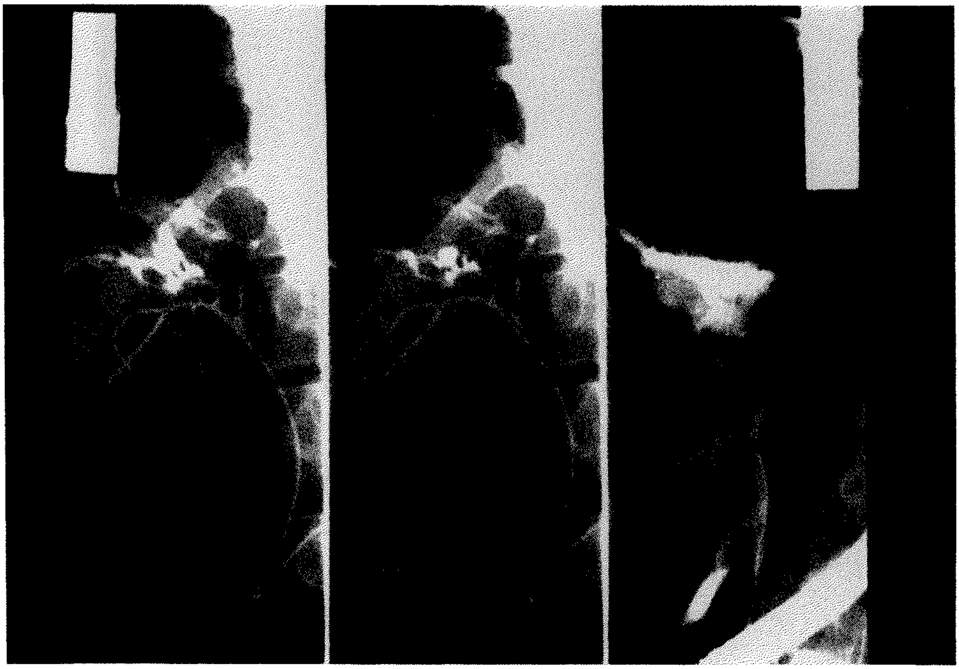

**Figure 2** Endoscopic retrograde cholangiogram on the 30th postoperative day. It revealed three leaks from the right and one from the left hepatic duct, as well as a large residual cavity.

drain in the cavity was closed and one week later it was removed. Cholangiograms performed through the catheter, demonstrated a steady reduction in size of the intrahepatic cavity. Twenty days after its placement, the catheter was periodically disconnected from suction and after a last cholangiogram, showing no residual cavity (Figure 4), it was closed for four days and then it was removed. This was the 94th postoperative day. The patient was discharged in a satisfactory condition, and 18 months later remains in good health, without signs of recurrence of the disease at recent ultrasound examination of the liver.

### DISCUSSION

Biliary cutaneous fistulae represent one of the major complications of surgery for hepatic hydatidosis. Barros reports a 3.8% incidence of external biliary fistulae in a series of 212 patients[6]. High pressure in the biliary system is a reason for persistence of such a fistula and endoscopic sphincterotomy, by reducing intraductal pressure, has been reported to result in healing[7,8].

Endoscopic biliary drainage by insertion of an endoprosthesis, is a technique originally applied for decompression in patients with malignant[8,9] or benign obstruction[10] of the biliary system. It has also been used successfully in the

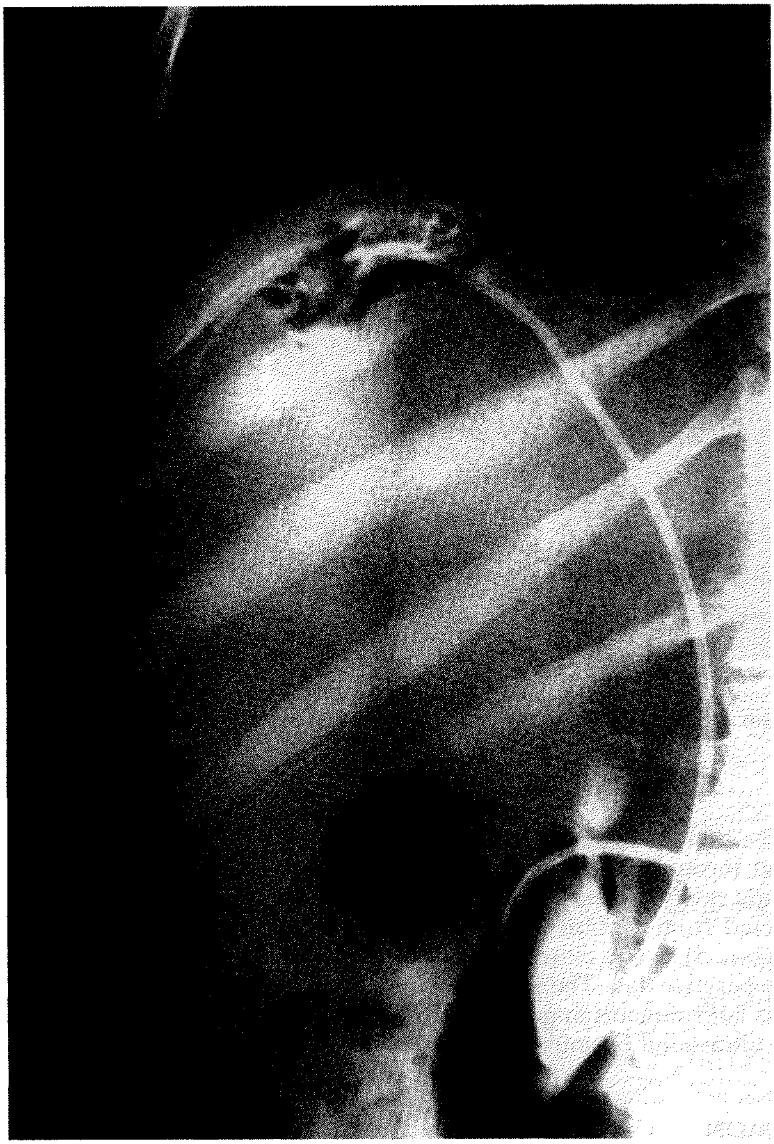

**Figure 3** A nasobiliary tube is selectively inserted in the right hepatic duct on the 70th postoperative day.

treatment of persistent biliary fistulae, caused by intraoperative trauma in the biliary tract or even external abdominal trauma[8,11,12,13].

Our patient had a biliary cutaneous fistula draining an exceptionally large amount of bile, originating from both the right and the left hepatic bile ducts. An endoscopic retrograde cholangiogram revealed no peripheral obstruction and the

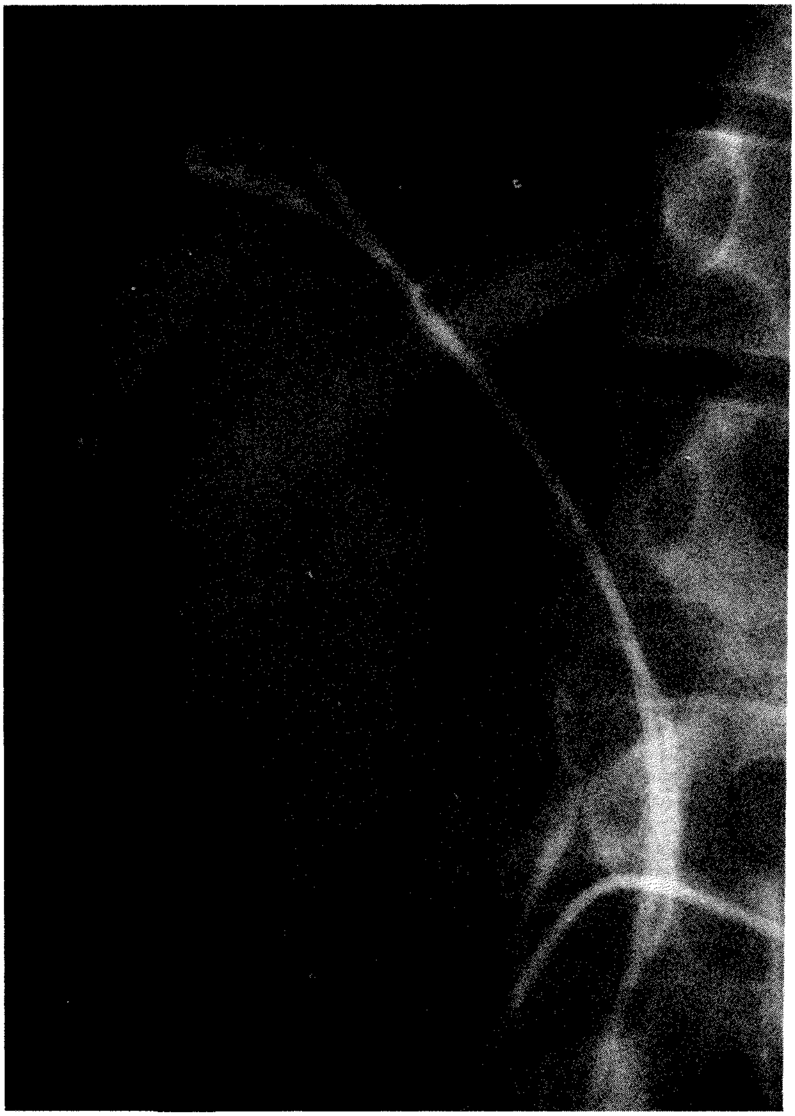

**Figure 4** The cavity has completely disappeared after 38 days of nasobiliary drainage. The tube was removed four days later.

sphincterotomy resulted in a significant but incomplete reduction in biliary drainage and closure of the leak from the left duct. A nasobiliary tube was then inserted selectively into the right hepatic duct to bypass the leaks, to decompress the biliary system and to allow better drainage of the bile into the duodenum. It was also believed that by applying a low negative pressure to the tube, the residual cavity would drain adequately into the bile duct and the duodenum, avoiding fluid collection, and allowing the regenerating liver to obliterate the cavity. This was documented by serial cholangiograms, the convenience of obtaining cholangiograms through the nasobiliary tube is another advantage of this technique. Delineation of the biliary system is thus achieved at any time and the progress of the treatment can be closely followed.

We have reported the successful treatment of an exceptionally high volume postoperative biliary cutaneous fistula, by nasobiliary drainage. We would consider this technique as being of potential benefit in patients with biliary fistulae not responding to expectant management or endoscopic sphincterotomy.

### *References*

1. Langer, J.C., Rose, D.B., Keystone, J.S., Taylor, B.R. and Langer, B. (1984) Diagnosis and management of hydatid disease of the liver: A 15-year North American experience. *Ann. Surg.*, **199**, 412–417
2. Del Olmo, L., Merono, E., Moreira, V.F., Garcia, T. and Garcia-Plaza, A. (1988) Successful treatment of postoperative external biliary fistulas by endoscopic sphincterotomy. *Gastrointest. Endosc.*, **34**, 307–309
3. Ponchon, T., Bory, R. and Chavaillon, A. (1987) Endoscopic retrograde cholangiography and sphincterotomy for complicated hepatic hydatid cyst. *Endoscopy*, **19**, 174–177
4. Goldin, E., Katz, E., Wengrower, D., Kluger, Y., Haskel, L., Shiloni, E. and Libson, E. (1990) Treatment of fistulas of the biliary tract by endoscopic insertion of endoprosthesis. *Surg. Gynecol. Obstet.*, **170**, 418–423
5. Krige, J.E.J., Bornman, P.C., Beningfield, S.J., Nieuwoudt, J.H.M. and Terblanche, J. (1990) Endoscopic embolization of external biliary fistulae. *Br. J. Surg.*, **77**, 581–583
6. Barros, J.L. (1978) Hydatid disease of the liver. *Am. J. Surg.*, **135**, 597–600
7. Vignote, M.L., Mino, G., de la Mata, M., de Dios, J.F., Gomez, F. (1990) Endoscopic sphincterotomy in hepatic hydatid disease open to the biliary tree. *Br. J. Surg.*, **77**, 30–31
8. Cairns, S.C., Heagerty, A., Dowsett, J.F., Vaira, D., Polydorou, A.A. and Hatfield, A.R.W. (1988) Successful endoscopic management of post-operative bile leak. *Gut*, **29**, A 1489
9. Huibregste, K. and Tytgat, G.N. (1982) Palliative treatment of obstructive jaundice by transpapillary introduction of large bore bile duct entoprosthesis. *Gut*, **23**, 371–375
10. Siegal, J.H., Harding, G.T. and Chateau, F. (1982) Endoscopic decompression and drainage of benign and malignant biliary obstruction. *Gastrointest. Endosc.*, **28**, 79–82
11. Goldin, E., Lobson, E. and Rachmilewitz, D. (1987) Endoscopic insertion of endoprostheses in the treatment of postoperative cutaneous biliary fistula. *Surgery*, **102**, 88–90
12. Sauerbruch, T., Weinzierl, M., Holl, J. and Pratschke, E. (1986) Treatment of postoperative bile fistulas by internal endoscopic biliary drainage. *Gastroenterology*, **90**, 1998–2003
13. Smith, A.C., Schapiro, R.H., Kelsey, P.B. and Warshaw, A.L. (1986) Successful treatment of nonhealing billiary-cutaneous fistulas with biliary stents. *Gastroenterology*, **90**, 764–769

(Accepted by S. Bengmark 15 January 1992)

### INVITED COMMENTARY

This case nicely illustrates once again that a non-healing external biliary fistula following an operation upon the biliary tract can be encouraged to heal by reducing the "vis-a-tergo" or pressure from behind which forces the drainage to continue. The factors which contribute to the non-healing include the volume of bile produced in that segment of the system, the size of the defect in biliary integrity, and the outflow pressure. As noted by the authors, endoscopic sphincterotomy has been used successfully to address the latter factor, but that solution may be inadequate, as in the present case. Endoscopic or percutaneous stenting may be particularly useful when the leak is in the extrahepatic portions of the bile duct because the stent bridges the defect and allows closure in a dry field, as well as channeling the bile flow[1]. When the leak is peripheral, as in the present case, as nasobiliary catheter can more easily be placed out to the region of the injury and negative pressure applied to aspirate bile away from the point of leak. As a cautionary note, this type of non-operative approach is only appropriate when the biliary-cutaneous fistula is well-contained and the patient does not have signs of sepsis or bile peritonitis. In those other circumstances, reoperation for better external drainage should be mandatory, although internal biliary drainage may still be a useful complementary maneuver.

*Reference*

1. Smith, A.C., Schapiro, R.H., Kelsey, P.B. and Warshaw, A.L. (1986) Successful treatment of non-healing biliary-cutaneous fistulas with biliary stents. *Gastroenterology*, **90**, 764–769

Andrew Warshaw
Harvard Medical School
Boston, Mass. 02117, USA

### INVITED COMMENTARY

Postoperative bile leakage with internal and external fistulae is an infrequent but serious complication of biliary tract surgery[1,2]. Usually these leaks are due to inadvertent trauma to the biliary tree or to inadequate closure of the cystic stump. Fistulae may also develop following surgical cyst drainage in hydatid disease when the cyst has open communication with the biliary tree[3]. Obstruction of bile flow in the distal common bile duct by stones or strictures may add to bile leakage and may prevent spontaneous closure.

These patients present early in the post-operative phase with an uncomplicated external fistula or with peritonitis, sepsis and intra-abdominal abscesses due to internal leakage[4,5].

Most fistulae of the gastrointestinal tract heal spontaneously when the flow of fluids is diminished or diverted. Therefore also biliary fistulae are best managed by lowering the biliary pressure and by facilitating the bile flow towards the duodenum. Although this can be achieved surgically, this is not an attractive option in the acute phase because of its considerable morbidity and mortality[6,7]. Non-surgical treatment modalities should therefore be applied. The percutaneous transhepatic route has been successfully used in this setting, but is attended with a high complication rate and is especially unattractive when the bile ducts are not dilated[8–11]. The percutaneous approach should only be used if the endoscopic approach has failed. The endoscopic procedures include endoscopic sphincterotomy, stone extraction, naso biliary drainage or stent insertion[12–16].

Most uncomplicated external fistulae close spontaneously. If the fistula does not close direct cholangiography is mandatory to rule out distal strictures or stones.

Endoscopic sphincterotomy and stone extraction or an endoscopic biliary endoprosthesis is then indicated. Endoscopic sphincterotomy promotes healing of the fistula when no distal obstruction is present. Patients who develop infectious complications should have immediate endoscopic treatment. Endoscopic sphincterotomy should also be performed even when no distal obstruction is present to speed up fistula closure. Surgical or percutaneous abscess drainage may be indicated. Once abscess formation has occurred, the prognosis for these patients depends no longer on the successful closure of the biliary fistula, but more on the successful drainage of the abscesses.

There is uncertainty with respect to the recommended time of waiting for spontaneous closure in patients with a non-complicated fistula. Furthermore it is difficult to predict which patient will develop infectious complications. We advocate early ERCP with antibiotic coverage in all patients with biliary leaks mainly to diagnose distal obstruction or leaks from the common bile duct proper. Early endoscopic treatment may then prevent the development of infectious complications from ongoing leakage. A biliary stent should only be inserted in patients with bile duct strictures or common bile duct trauma to bridge the opening. In all other instances the result of endoscopic sphincterotomy alone should first be awaited. In case of ongoing leakage one may proceed to the insertion of a stent or a naso biliary catheter.

In this issue Vagianos *et al.*[17] describe the successful endoscopic treatment of a patient with a high output external biliary fistula following surgical hydatid cyst drainage. Usually these fistulae heal spontaneously following endoscopic sphincterotomy by which biliary pressure is lowered. Adhering to the main principles for treatment of fistulae a nasobiliary catheter was inserted to aspirate bile and further lower the bile pressure. This resulted in closure of the fistula. The authors clearly demonstrate the benefits of endoscopic procedures in these difficult post-operative biliary complications.

### *References*

1. Hillis, T.M., Westbrook, K.C., Caldwell, F.T. and Read, R.C. (1977) Surgical injury of the common bile duct. *Am. J. Surg.*, **134**, 712–716
2. Hadjis, N.S., and Blumgart, L.H. (1988) Injury to segmental bile ducts. *Arch. Surg.*, **123**, 351–353
3. Barros, J.L. (1978) Hydatid disease of the liver. *Am. J. Surg.*, **135**, 597–600
4. Collins, P.G. and Gorey, T.F. (1984) Iatrogenic biliary stricture: presentation and management. *Br. J. Surg.*, **71**, 900–902
5. Sandberg, A.A., Johansson, B. and Bengmark, S. (1985) Accidental lesions of the common bile duct at cholecystectomy. II, Results of treatment. *Ann. Surg.*, **209**, 452–455
6. Martin, J.K., Van Heerden, J.A. (1980) Surgery of the liver, biliary tract and pancreas. *Mayo Clin. Proc.*, **55**, 333–337
7. McSherry, C.K., and Glenn, F. (1980) The incidence and causes of death following surgery for non-malignant biliary tract disease. *Ann. Surg.*, **191**, 271–275
8. Smith, A.C., Shapiro, R.H., Kelsey, P.B. and Warshaw, A.L. (1986) Successful treatment of nonhealing biliary-cutaneous fistulas with biliary stents. *Gastroenterology*, **90**, 764–769
9. Sauerbruch, T., Wienzierl, M., Holl, J. and Pratschke, E. (1986) Treatment of postoperative bile fistulas by internal endoscopic drainage. *Gastroenterology*, **90**, 1998–2003
10. Zuidema, G.D., Cameron, J.L., Sitzmann, J.V., Kadir, S., Smith, G.W., Kaufman, S.L. and White, R.I. (1983) Percutaneous transhepatic management of complex biliary problems. *Ann. Surg.*, **197**, 584–593
11. Speer, A.G., Cotton, B.P., Russell, R.C.G., Mason, R.R., Hatfield, A.R.W., Leung, J.W.C., MacRae, K.D., Houghton, J. and Lennon, C.A. (1987) Randomized trial of endoscopic versus percutaneous stent insertion in malignant obstructive jaundice. *Lancet*, **II**, 57–62
12. O'Rahilly, S., Duignan, J.P., Lennon, J.R. and O'Malley, E. (1983) Successful treatment of a postoperative external biliary fistula by endoscopic papillotomy. *Endoscopy*, **15**, 263–268
13. Huibregtse, K. (1988) Endoscopic biliary and pancreatic drainage. Stuttgart, New York: Thieme Verlag, pp. 68–73
14. Del Olmo, L., Meroño, E., Moreira, V.F., Garcia, T. and Garcia-Plaza, A. (1988) Successful treatment of postoperative external biliary fistulas by endoscopic sphincterotomy. *Gastrointest. Endosc.*, **34**, 307–309
15. Ponchon, T., Gallez, J.F., Valette, P.J., Chavaillon, A. and Bory, R. (1989) Endoscopic treatment of biliary tract fistulas. *Gastrointest. Endosc.*, **33**, 490–498
16. Davids, P.H.P., Rauws, E.A.J., Coene, P.P.L.O., Tytgat, G.N.J. and Huibregtse, K. (In press) Postoperative bile leakage: the endoscopic management
17. Vagianos, C., Polydorou, A., Karatzas, T., Vagenas, C. and Stavropoulos, M. (1992?) Successful treatment of postoperative external biliary fistula by selective nasobiliary drainage. *HPB Surg.* (1991) (this issue)

K. Huibregtse
Department of Gastroenterology
University of Amsterdam
Academic Medical Center
Meibergdreef 9
1105 AZ Amsterdam, The Netherlands

### INVITED COMMENTARY

Biliary cutaneous fistulas follow loss of ductal integrity and persistence is related to existing pressure gradients within the biliary system. Under physiologic conditions a pressure gradient is maintained between the biliary system and the duodenum by the sphincter of Oddi. An increasing basal pressure gradient is found in sequence between duodenum, ampulla and the biliary system. In the presence of a competent and functioning sphincter mechanism, bile follows the path of least resistance along a fistulous tract. Endoscopic sphincterotomy abolishes the pressure gradient and facilitates bile flow into the duodenum and allows fistula closure.

Intrahepatic biliary fistulas may present complex management decisions and are encountered after blunt or penetrating liver trauma, drainage of amoebic or pyogenic liver abscesses or after evacuation of hydatid cysts with a pre-existing communication with the biliary system. In the majority of cases, spontaneous closure of the fistula occurs, provided no distal obstruction is present. Intervention is required only where the fistulous tract shows no dimunition in flow or persists for longer than two weeks. Prolonged external bile loss may lead to substantial fluid and electrolyte depletion. In addition, chronic external biliary fistulas may be complicated by steatorrhoea, calcium and vitamin D malabsorption, infection and poor wound healing[1,2].

Surgery has previously been the standard therapy for persistent biliary cutaneous fistulas. Reoperation may, however, be complex because of scarring, infection, inadequate exposure and difficulty in delineating the site of extravasation[2]. Endoscopic treatment is now the treatment of choice in patients with persistent or large volume biliary cutaneous fistulas. The technique provides a rapid and safe non-operative method of treatment in often critically ill patients. Endoscopic retrograde cholangiography provides accurate and precise visualization of the fistula site, especially in intrahepatic lesions. Cholangiographic factors which require evaluation in chronic fistulas are the presence of a distal biliary obstruction, the extent of the duct injury, and the exact location and number of fistulas. In addition, ultrasonic detection and percutaneous drainage of associated subhepatic or subphrenic collections or abscesses is an essential element in management.

We use a progressive sequence of endoscopic intervention in persistent biliary fistulas. An adequate endoscopic sphincterotomy should first be performed to provide adequate biliary decompression by equalizing intrabiliary and duodenal pressure gradients and the response evaluated. If there is no satisfactory dimunition in fistula volume, an endoscopic biliary stent should be placed. Larger diameter endoprostheses allow increased bile flow with longer stent patency and fewer occlusions. In extrahepatic bile duct fistulas, the stent has the additional advantage of bridging the fistula orifice at the site of extravasation. An alternative to stenting is selective nasobiliary tube placement proximal to the fistula with constant tube suction. Nasobiliary catheters have the advantage of being easy to flush if occluded. On the other hand, an internal endoprosthesis is better tolerated and avoids bile loss. The optimal period of stenting is debatable, but four to six weeks seems a reasonable period. In the unusual event of neither these techniques being effective, endoscopic embolization of the fistulous tract or the draining peripheral duct with a Gelfoam pledget has been used[3]. Endoscopic intervention with sphincterotomy, biliary stenting or selective nasobiliary drainage in persistent biliary fistulas has eliminated the need for complex re-operations and is the therapeutic procedure of choice in this difficult problem.

J.E.J. Krige
Department of Surgery
University of Cape Town
Observatory 7925
Cape Town, South Africa

### *References*

1. Hoffman, B.J., Cunningham, J.T. and Marsh, W.H. (1990) Endoscopic management of biliary fistulas with small calibre stents. *Am. J. Gastroenterol.*, **85**, 705–707
2. Smith, A.C., Shapiro, R.H., Kelsey, P.B. and Warshaw, A.L. (1986) Successful treatment of nonhealing biliary-cutaneous fistulas with biliary stents. *Gastroenterology*, **90**, 764–769
3. Krige, J.E.J., Bornman, P.C., Benningfield, S.J., Nieuwoudt, J.H.M., and Terblanche, J. (1990) Endoscopic embolization of external biliary fistulae. *Br. J. Surg.*, **77**, 581–583

